# Interleukin-10 polymorphisms in Spanish IgA deficiency patients: a case-control and family study

**DOI:** 10.1186/1471-2350-7-56

**Published:** 2006-06-27

**Authors:** Javier Ortiz, Miguel Fernández-Arquero, Elena Urcelay, Raquel López-Mejías, Antonio Ferreira, Gumersindo Fontán, Emilio G de la Concha, Alfonso Martínez

**Affiliations:** 1 Clinical Immunology Department, Hospital Clínico San Carlos, Madrid; 2 Immunology Section, Hospital Universitario La Paz, Madrid

## Abstract

**Background:**

IgA deficiency (IgAD) is the most common primary immunodeficiency in Caucasians. Genetic and environmental factors are suspected to be involved in the development of the disease. Interleukin-10 (IL-10) is a cytokine with stimulatory activity on immunoglobulin production and it may be an important regulator in IgAD pathogenesis. The IL-10 gene contains several single nucleotide polymorphisms (SNPs) and two polymorphic microsatellites located in the 5'-flanking region. Our aim was to ascertain if any of these polymorphic markers are associated or linked to IgAD in Spanish patients.

**Methods:**

We genotyped 278 patients with IgAD and 573 ethnically matched controls for the microsatellites IL-10R and IL-10G and for three single nucleotide polymorphisms at positions -1082, -819 and -592 in the proximal promoter of the gene. We also included in this study the parents of 194 patients in order to study the IL-10 haplotypes transmitted and not transmitted to the affected offspring.

**Results:**

The only allele where a significant difference was observed in the comparison between IgA deficiency patients and controls was the IL-10G12 allele (OR = 1.58 and p = 0.021). However, this p value could not withstand a Bonferroni correction. None of the IL-10R or promoter SNP alleles was found at a different frequency when patients were compared with controls.

**Conclusion:**

Our data do not show any significant difference in IL-10 polymorphism frequencies between control and IgAD patient samples. Their haplotype distribution among patients and controls was also equivalent and therefore these microsatellites and SNPs do not seem to influence IgAD susceptibility.

## Background

IgA deficiency (IgAD) is the most common primary immunodeficiency in Caucasians and is sometimes associated with deficiency of IgG2, IgG3, IgG4 and IgE [1]. Genetic studies indicate that IgAD may have an inherited component and the disease is associated with certain susceptibility genes located in the major histocompatibility complex region [2,3]. Celiac disease and selective IgA deficiency are frequently associated, and share a similar genetic background [4,5]. Environmental factors also play a role in the occurrence of IgAD. The most usual consequence of this disease is an increased susceptibility to infection [6].

Although the basic immunologic defect that underlies IgAD is unknown, a number of *in vitro *immunological alterations have been identified: arrest in the B cell differentiation pathway [7], and a decrease in T helper lymphocyte function [8,9].

IL-10 is a regulatory cytokine that has several functions; it is a potent stimulator of NK cells [10] and has stimulatory effects in B-cell proliferation but due to what has been termed "the dual role" of IL-10, it has also inhibitory effects and suppresses the proinflammatory functions of antigen-presenting cells (APCs) by antagonizing expression of costimulatory molecules, the release of proinflammatory cytokines and, in general, APC maturation [11-14]. In addition to inhibiting APC maturation, IL-10 prolongs their capability for antigen uptake while simultaneously postponing their migration to draining lymph nodes. Finally, the IL-10 knockout mouse develops chronic enterocolitis [15], highlighting its important role as modulator of mucosal immunity.

Peripheral blood mononuclear cells (PBMC) from IgAD patients cultured in the CD40/SAC (*Staphylococcus aureus *Cowan) system produced IgM and IgG, but not IgA. The addition of IL-10 to the cultures enhanced the production of IgM and IgG and most strikingly induced the production of high amounts of IgA. Thus, IL-10 can remove the block in B cell differentiation and allows B cells from IgAD patients to differentiate into IgA secreting cells [16].

The IL-10 gene is located on the long arm of human chromosome 1 and its proximal promoter contains three single nucleotide polymorphisms (SNPs) at positions -1082 (A/G), -819 (T/C) and -592 (C/A) (17–20). Only three haplotypes have been described in Caucasian populations: ATA, ACC and GCC. The production level of this cytokine is mainly determined by the mRNA synthesis rate, which in turn depends on these promoter polymorphisms [17]. The 5'-flanking region also contains two microsatellites: IL-10G and IL-10R [18,19]. The haplotypes formed by both microsatellites also correlate with cytokine production, presumably due to the linkage disequilibrium between promoter and microsatellite alleles forming extended haplotypes [20,21].

There is only a previous study analyzing polymorphisms of IL-10 polymorphisms in IgA deficiency [22], but the small sample size did not allow reaching definite conclusions. The present study is aimed at performing a comprehensive association analysis of IL-10R and IL-10G microsatellites and IL-10 proximal promoter SNPs in a large Spanish sample of IgA deficiency patients. We found no evidence of association of IL-10 polymorphisms to IgAD in the Spanish population in our study.

## Methods

The study group consisted of 278 Caucasian Spanish patients (45% female) with IgA deficiency. They were diagnosed by serology criteria (serum IgA level below 0.05 g/l). The samples were recruited from a single centre (La Paz Hospital, Madrid, Spain) and most of them have been included in previous studies from our group. Informed consent was obtained from all the participants. This study was approved by the Ethics Committee of the Hospital Clínico San Carlos, and it is in compliance with the Helsinki declaration. The patients can be distributed in different groups, according to their clinical symptomatology: allergy (16%), autoimmune disease (17%), celiac disease (6%), infectious diseases (13%), psoriasis (4%), other (44%) or according to the presence of auto-antibodies: anti IgA antibodies (16%), antinuclear antibodies (11%), anticardiolipins (3%), microsomal antibodies (3%) and no antibodies (67%). We could also obtain progenitor samples of 194 patients (usually trios father-mother-patient). A group of 573 healthy white, unrelated subjects (50.8% women) from the Madrid region (mainly Hospital employees and blood donors) was selected as controls. Many of the patient and familiy samples have been included in previous studies of our group in the field of IgAD genetics [23-25]

IL-10G and IL-10R microsatellites were amplified using primers and conditions previously described [20]. Samples were subsequently denatured and run on an ABI Prism 3100 automatic sequencer (Applied Biosystems, Foster City, Calif.). Each sample included an internal size standard (HD400 ROX; Applied Biosystems) to achieve a highly consistent measure. The results were analyzed using GeneMapper v3.0 (Applied Biosystems).

There are only three IL-10 promoter haplotypes (GGC, ATA, ACC) described [21,26,27] in our population. That is why we have only analyzed the first (-1082) and the third (-592) position in the IgAD patients and we then obtained the second position (-819) by a simple deduction. The SNP study has been performed using TaqMan probes (C___1747360_10 for -1082 and C___1747363_10 for -592) under conditions recommended by the manufacturer. The samples were read by two observers and some samples with known results were included in the analysis as quality controls. Healthy controls were typed (all three positions) as described elsewhere [26]. Concordance between the two techniques was total.

The frequencies of each marker allele in patients and controls were compared by a standard Chi-Square test. The comparisons between haplotypes transmitted and not transmitted were also done by this method. For each locus, a Bonferroni correction was applied. Only alleles found at a frequency higher than 3% were taken into account for correction purposes. A result was considered significant if P_c _< 0.05. The present study has a statistical power of 80% to detect an association with a relative risk of 1.5 for an allele with a phenotypic frequency of 0.2.

## Results

Table 1 shows the results from the IL-10R and IL-10G microsatellite analysis. The alleles found at a higher frequency were IL-10R2 for IL-10R, and IL-10G9 and IL-10G13 for IL-10G, as described in other Caucasian populations. The IL-10R1, IL-10G1-G6 and IL-10G16-G17 alleles were extremely rare, also in keeping with previous reports.

**Table 1:** Phenotypic frequencies of IL-10R and IL-10G microsatellites in patients with IgA deficiency (n = 278) and controls (n = 573)

IL-10R	Patients	%	Controls	%
2	266	96	552	96
3	97	35	198	34
4	11	4	19	3.3
				
IL-10G	Patients	%	Controls	%
7	13	5	16	3
8	12	4	34	6
9	150	54	305	53
10	37	13	75	13
11	66	24	140	24
12	47	17	63	11
13	109	39	262	46
14	44	16	81	14
15	7	3	18	3

In the comparison between IgA deficiency patients and controls the only significantly different value observed was the frequency of IL-10G12 allele, higher in patients, (OR = 1.58, p = 0.021). However, this p value could not withstand a Bonferroni correction. None of the IL-10R alleles was found at a different frequency in the group of patients when compared with controls.

Table 2 displays the results observed in IL-10 promoter polymorphisms. The IL-10 promoter haplotype with higher frequency, both in patients and in controls, was G(C)C. We did not observe any significant difference between patients and controls when promoter frequencies were compared.

**Table 2:** Phenotypic frequencies of IL-10 promoter haplotypes in patients with IgA deficiency (n = 271) and controls (n = 529)

Allele	Patients	%	Controls	%
ATA	121	45	235	44
ACC	150	55	287	54
GCC	179	66	332	63

Table 3 shows the extended haplotypes comprising IL10R, IL10G microsatellites and IL-10 gene promoter SNPs more frequently found in families, sorted in transmitted to the patients and not transmitted to them. The extended haplotype more frequent was IL10R2-IL10G9-A(T)A. Haplotypes extremely rare in our study are included under the "Others" heading. As it can be observed, there are more transmitted haplotypes in total (388) than non-transmitted (332). This is because in absence of one progenitor we could not obtain the haplotype non-transmitted by that progenitor, unless an additional family member was available. We can observe a greater proportion of IL10R2-IL10G13-G(C)C haplotype transmitted than no transmitted (OR = 2.0, p = 0.024), but again the p value failed to reach a statistically significant level after Bonferroni correction.

**Table 3:** IL-10R/IL-10G/promoter SNPs haplotypes, transmitted (388) and not transmitted (332)

Haplotype	T	%	NT	%
2/9/ATA	66	17	61	18
2/13/ACC	55	14	40	12
3/9/GCC	42	11	27	8
2/11/GCC	33	9	37	11
2/13/GCC	34	9	15	5
2/14/ACC	24	6	27	8
2/12/ACC	22	6	12	4
3/10/GCC	8	2	11	3
3/9/ATA	15	4	16	5
2/11/ACC	10	3	9	3
2/12/GCC	10	3	5	2
Other	69	18	72	22

Due to the well-known association of IgAD with HLA haplotypes (marked by DR1 or DRB1*0102, DR3 and DR7 as risk factors, and DR2-DQ6 as a protection haplotype), we performed HLA-stratified analyses of the IL-10 polymorphisms, but no interaction of IL-10 alleles with any predisposition-related HLA allele or haplotype was found (data not shown).

## Discussion

In this study we have undertaken an association analysis of IL-10 polymorphisms (IL-10R and IL-10G microsatellites and promoter SNPs) in a large sample of Spanish patients with IgA deficiency. Our data do not show any significant difference between control and patient samples. The haplotypic distribution among patients and controls was equivalent and consequently these microsatellites and SNPs do not seem to affect IgA deficiency susceptibility *per se*.

A possible caveat that has always to be considered when a negative result is reached is that the sample size may not be large enough and that in consequence we can not see a possible difference between cases and controls. It is our belief that the present study has a reasonable statistical power (80% power to detect a relative risk of 1.5 with an allele frequency of 0.1) so we can confidently conclude that if there is in fact an effect, it should be a rather small one because our sample is powered enough to detect any clinically relevant effect. Another reason that could explain our negative results is that the polymorphisms we have studied do not have functional relevance, or are not in linkage disequilibrium with relevant polymorphisms located elsewhere in the IL-10 gene. However there are functional studies implicating the position -1082 of the IL-10 promoter and IL-10R/IL-10G microsatellite haplotypes [20] in the production *in vitro *of IL-10. Moreover, the high degree of linkage disequilibrium and the presence of extended haplotypes [21] make our choice of genetic markers useful to incorporate most of the genetic information present at the IL-10 locus.

As we know, IL-10 has a dual role both as inhibitory and stimulatory component of the immune responses. There are not references in the bibliography reporting that an alteration of IL-10 levels could eventually cause an immunodeficiency. On the contrary, there have been many studies that find genetic associations of the IL-10 polymorphisms with inflammatory diseases like rheumatoid arthritis, type 1 diabetes, systemic lupus erythematosus or inflammatory bowel diseases [19,26-30]. Therefore the evidence suggests that IL-10 deficiency or alteration could be more important as an etiologic factor in autoimmune diseases than in immunodeficiencies. This conclusion is supported by studies with IL-10 knock-out mice, which develop a chronic enterocolitis. The administration of IL-10 to these mice ameliorates the symptoms of the disease [15]. Administration of recombinant human IL-10 to colitis patients is being evaluated as a therapeutic option in colitis patients. It may be speculated that the role of IL-10 as stimulatory factor is redundant with that of other cytokines (e.g. IL-6), and therefore a defect in one of its positive effects is not clinically detectable. Only inhibitory functions of IL-10 might be non-redundant.

Therefore we can conclude that, in terms of sample size, genetic markers included, and haplotype analysis, our work greatly expands previous bibliography about the effect of IL-10 polymorphisms on IgAD. It would be really interesting in the future to analyze further the IL-10 *locus *and its possible interactions with other *loci *coding for other molecules involved in B cell maturation and final differentiation to plasma cells, like genes coding for IL-10 receptor chains or the gene recently described as associated with IgAD and common variable immunodeficiency, TACI [31,32].

## Conclusion

Our data do not show any significant difference in IL-10 polymorphism frequencies between control and IgAD patient samples. Their haplotype distribution among patients and controls was also equivalent and therefore these microsatellites and SNPs do not seem to influence IgAD susceptibility.

## Competing interests

The author(s) declare that they have no competing interests.

## Authors' contributions

JO performed the genetic and statistical analyses and drafted the manuscript. MFA helped with data acquisition and coordinated the study. EU helped to design the study and revised the manuscript critically. RLM participated in data acquisition and revised critically the manuscript. AF participated in the collection of samples and IgA quantification and participated in the design and coordination of the study. GF participated in the collection of samples and IgA quantification. EGdC participated in the design of the study and revised critically the manuscript. AM conceived the study, participated in its design and coordination, data acquisition and revised critically the manuscript.

## Pre-publication history

The pre-publication history for this paper can be accessed here:


